# Interprofessional team-based collaboration between designated GPs and care home staff: a qualitative study in an urban Danish setting

**DOI:** 10.1186/s12875-023-01966-1

**Published:** 2023-01-04

**Authors:** Line Due Christensen, Linda Huibers, Flemming Bro, Morten Bondo Christensen, Anna Mygind

**Affiliations:** 1 Research Unit for General Practice, Bartholins Alle 2, 8000 Aarhus C, Denmark; 2 Department of General Practice, Institute for Public Health, Aarhus University, Bartholins Alle 2, 8000 Aarhus, Denmark

**Keywords:** General practitioners, Interdisciplinary communication, Physician-nurse relations, Primary health care, Qualitative research, Residential facilities, Denmark

## Abstract

**Background:**

Being a general practitioner for residents in many care homes may challenge communication with residents, relatives, and care home staff, and potentially lead to lower quality of care. Several countries have therefore introduced different solutions to reduce the number of general practitioners at each care home. In 2017, the designated general practitioner model was introduced at many Danish care homes. This study aimed to evaluate experiences from the interprofessional team-based collaboration between designated general practitioners and care home staff with regular contact with the designated general practitioners in an urban Danish setting.

**Methods:**

A qualitative design was applied using semi-structured interviews. Eight interviews (three group interviews and five individual interviews) were conducted with four designated general practitioners and seven care home staff members at four care homes in an urban setting of Central Denmark Region, Denmark. The interviews were transcribed verbatim, and data were analysed using content analysis with inspiration from the theory of relational coordination. The study followed the guidelines addressed in the COREQ (Consolidated Criteria for Reporting Qualitative Research) framework.

**Results:**

The initiation of the designated general practitioner model was experienced to contribute to more clear, precise, and timely communication between care homes and the general practitioner. An improved mutual acknowledgement of roles and competencies was experienced between designated general practitioners, care home nurses, and sometimes also social and health care assistants. The more frequent visits by the general practitioners at the care homes, as a result of the designated general practitioner model, resulted in more face-to-face communication between care home staff and designated general practitioners. Professional differences in the interpretation of the patient’s needs were still present, which at times caused a frustrating compromise of own professional competencies. An important reason for the overall perception of improved collaboration was attributed to the more frequent dialogue in which the care homes staff and the designated general practitioners exchanged knowledge that could be applied in future patient encounters.

**Conclusion:**

The designated general practitioner model implied an improved collaboration between general practitioners and care homes staff. Clear, precise, and timely communication between care homes and the general practitioners, as well as mutual trust and acknowledgement was experienced to be essential for the collaboration. An important reason for the overall perception of an improved collaboration was attributed to the more frequent dialogue (more frequent general practitioner visits at the care homes) in which the care homes staff and the designated general practitioners exchange knowledge which again could be applied in future patient encounters.

**Supplementary Information:**

The online version contains supplementary material available at 10.1186/s12875-023-01966-1.

## Background

The steadily increasing number of older people presents new challenges for health care systems [1]. Older people admitted to care homes represent a frail population, who are weakened cognitively and physically [2, 3]. They use health care services more frequently [4] and experience greater comorbidity and disease complexity compared with the general older population [3, 5].

In many countries, care home residents retain their regular general practitioner (GP), who treated them before being admitted to the care home. This may present some challenges, such as long-distance between the GP and the care home, GP’s lack of knowledge of the care home staff, and many GPs visiting the same care home. In Germany, on average, 23 GPs visit a single care home. Together with the nurses’ working shift pattern, this results in a wide variety of collaboration patterns [6]. Poor communication between patients, relatives, GPs, and care home staff may result in negative health-related outcomes, such as avoidable hospitalizations [7, 8].

Countries have introduced different solutions to reduce the number of GPs at each care home. The Netherlands has care home physicians employed by the care homes [9], whereas care homes in Canada, Germany, and Norway have a designated GP in each care home, who provides primary care for all residents [10]. In Denmark, implementation of the designated GP model started in 2017, but not all care homes have yet a designated GP affiliated. Furthermore, since a designated GP is not mandatory, some residents have other GPs [11]. Introducing the designated GPs at Danish care homes has been shown to reduce the number of hospitalisations [12].

To be able to use the already existing leverage with the designated GP model [13], it is important to understand the mutual experiences of the team-based collaboration between care home staff and the designated GP in the newly started designated GP model. Thus, we aim to evaluate the interprofessional team-based collaboration, as experienced by designated GPs and care home staff in Danish care homes in an urban Danish setting.

## Methods

### Design

We conducted a descriptive qualitative study, using semi-structured interviews to gain insights into designated GPs’ and care home staffs’ perspectives of their mutual interprofessional collaboration. We used content analysis with inspiration from the theory of relational coordination [14]. To secure the quality and trustworthiness of the study, we followed the main domains and each sub-item presented in the checklist: The Consolidated criteria for reporting qualitative research (COREQ) 32-item checklist (Additional file 1) [15].

### Setting

Denmark has a public tax-financed health care system, which provides free and equal access to treatment for primary care and hospitals, for all people. The GP acts as a gatekeeper and first-line provider; meaning that a referral from a GP is required for most office-based specialists and in- and outpatient hospital treatment [16]. Frail individuals, who need all-day care, are eligible for care home residency according to Danish law. It is, however, up to the local municipalities to appoint residency to individuals with the greatest need, based on functional capacity [17]. In Denmark, around 40,000 older adults (≥ 65 years old) live in care homes [18]. Residents in care homes can maintain the GP they had before entering the care home unless they move to a care home too far away from the GP to perform home visits or they actively choose another GP. The health professionals in Danish care homes usually include nurses (42 months of education), social and health care assistants (32 months of education), social and health care helpers (14 months of education), occupational therapists (42 months of education), and physiotherapists (42 months of education). Social and health care assistants and -helpers constitute about 74% of the care home staff on duty during daytime on weekdays [19].

In 2017, the designated GP model was introduced at many Danish care homes [11]. In each care home, a single or a few GPs are assigned to be the designated GP while still maintaining their private practice. Residents are encouraged, but not obligated, to select the designated GP. No formal requirements exist regarding the organization of the collaboration between GPs and care homes, e.g. how often the GP must visit the care home and whether the GP provides teaching to the care home staff on topics related to the treatment of the residents [11]. Both nurses and social and health care assistants could be a part of the team-based collaboration with the designed GPs.

The study took place in an urban municipality in Denmark. The municipality is a part of the Central Denmark Region and has a population of about 350,000 inhabitants). At the time of the study, 46 out of 50 care homes in the municipality were affiliated with a designated GP.

### Participants

Care homes were eligible if they were affiliated with a designated GP. The care homes were purposefully selected in order to include care homes with a maximum variation regarding the organisation of the collaboration between care homes and the designated GP (from a questionnaire survey in a previous study [20]). LDC invited designated GPs and care home staff members who engaged in team-based collaboration with each other from the selected care homes to participate in an interview by email or telephone. In both the telephone- and the email messages the content was the same and included a formal invitation for the interview. In order to iteratively guide an adequate sample size, we used the concept of information power suggesting that the more information the sample holds, the lower number of participants is required [21]. After interviews with care home staff and GPs from four different care homes, we made an iterative interpretative judgement that there was sufficient information power to address study aims. In this process, we considered the study aim, sample specificity, use of established theory, analysis strategy, and quality of dialogue [21]. Our study aim was narrow due to a research question which was highly focused on the experience of the interprofessional team-based collaboration between the designated GPs and care home staff in Danish care homes. The participants were selected using purposive sampling for characteristics highly specific to the study aim. The theory of relational coordination informed both the interview guide and the analysis. As the interviewer was a researcher with previous qualitative experiences and the interview guide was developed by multiple rounds of discussions in the research group, we estimated the quality of dialogue to be sufficient.

### Data collection

The informants were affiliated with four different care homes with different organizations of team-based collaboration, thereby achieving a high level of information power [21, 22]. We invited four designated GPs and seven care home staff members (six nurses and one social and health care assistant) and all accepted to participate in an interview. We conducted eight face-to-face interviews (three group interviews and five individual interviews) (Table 1). The group interviews were conducted with care home staff who worked as a team from the same care home.

**Table 1:** Overview of informants

Care home	Interview type	Profession	Gender	Informants
A	Group interview	Nurse	Female	N1
		Social and health care assistant	Female	HA1
	Single interview	Designated GP	Female	GP1
B	Group interview	Nurse	Female	N2
		Nurse	Female	N3
	Single interview	Designated GP	Male	GP2
C	Group interview	Nurse	Female	N4
		Nurse	Female	N5
	Single interview	Designated GP	Female	GP3
D	Single interview	Nurse	Female	N6
	Single interview	Designated GP	Female	GP4

Interviews were conducted in October–December 2019 at care homes (care home staff) or GP private practices (GPs). The interviews were based on a semi-structured interview guide with open-ended questions based on the theory of relational coordination [23, 24]. Relational coordination is defined as coordinating work through relationships of shared goals, shared knowledge, and mutual respect [23, 24]. The interview guide covered six topics: background information, tasks, significance of the designated GP model, GPs teaching at the care home, collaboration between GPs and care home staff, and recommendations to improve the collaboration (see Additional file 2). The interview guide was developed during multiple rounds of discussion between LDC (MSc Pharm), postdoc), AM (MSc (Public Health), senior researcher), and LH (MD, senior researcher), and was continuously refined throughout the process of data collection. LDC performed all interviews, which lasted 24–55 minutes. All participants received oral information about the study before participating. LDC wrote field notes immediately after each interview. A research assistant transcribed the audio-recorded interviews verbatim, and LDC checked the transcripts for fidelity.

### Data analysis

All transcripts and field notes were read and coded independently by LDC, focusing on the overall aim of the study. Key data units were identified and coded through an iterative process, in which the coding list was continuously developed and refined. Once the coding process was complete, codes with mutual characteristics were grouped into emergent themes that were finally assembled into the final themes of the theory of relational coordination [23, 24] and the overall aim of the study. Thus, data were analysed both inductively and deductively in the process. All authors engaged in ongoing discussions about and agreed upon the emergent themes. No coding software was used.

## Results

Two main themes appeared from the analysis: ‘Acknowledging competencies and roles’ and ‘Interprofessional communication’.

### Acknowledging competencies and roles

#### Mutual trust, understanding, and knowledge about professions

A trusting team-based relationship between the designated GP and the care home staff was perceived to benefit the collaboration. GPs and care home staff illustrated this by underscoring the importance of trust in the interprofessional team-based collaboration. A nurse said:

*“I certainly have the feeling that they [GPs] think that our professional competency is OK and that they can rely on what we say. And I trust what they say. So, it is a collaboration. It is like at the hospital; the doctor cannot do his or her job if a nurse or an assistant has not done their part. Well, you depend on one another in that way. So I actually feel that they trust us.”* (N2)

The designated GPs experienced that the nurses’ professional competencies were of high quality and that they trusted nurses as a professional and their professional judgment. A designated GP exemplified how a nurse could assess the patient’s need, and if it was relevant to involve the designated GP, the nurse would provide sufficient information:

*“And it’s also because she [the nurse] is good at finding out […] where there is a need for a medical specialist or rather a need for a nursing specialist, or nursing care, right. And if there is something that she finds that she needs my help for, then she is really good at writing a good correspondence that makes it possible for us to move on with it.”* (GP3)

Some designated GPs experienced that a close one-on-one collaboration between a designated GP and a nurse was important. A designated GP explained:

*“I believe that it really makes a difference when we [GPs] sit and talk with the nurses about treatment, and we wonder about different problematic issues. And we tell them how to treat different things”.* (GP2)

Thus, mutual trust, understanding, and knowledge about professions, education level, and individual competencies were experienced to improve as the designated GP and the care home staff got to know each other. Thereby, the collaboration and the interprofessional communication improved.

#### Acknowledgement of the different roles

A nurse experienced that the designated GPs sensed that the nurses had become ‘gatekeepers’ in the designated GP model:

*“I find that the doctors [GPs] express that they feel that some kind of ‘gatekeeper’ has now been introduced. Like, we are a kind of intermediate station, where we filter out the minor things – that then are dealt with in-house – and that the need for care is first assessed; Is it something that can wait, or is it something that must be dealt with now? Is it something that should be written down in a correspondence [to the GP]?”* (N1)

However, some designated GPs expressed that it was frustrating if this ‘gatekeeping’ did not function as intended. Both care home staff and designated GPs discussed whether the interprofessional communication about patient-related issues should take place between only GPs and nurses or also between GPs and social and health care assistants. GPs experienced too many unnecessary contacts, particularly from the social and health care assistants, and some GPs highlighted that the regular contact should be with the nurses and not the social and health care assistants. A GP explained how the interprofessional communication in the care home she was affiliated to, had developed since the initiation of the designated GP model:

*“In the beginning, assistants and social and health care assistants used to participate in the ward rounds, and that did not work. They also made a lot of irrelevant enquiries; things that they should first have cleared with a nurse.”* (GP1)

A nurse described how the social and health care assistants experienced the situation in their care home when the designated GP rejected to communicate directly with the social and health care assistants:

*“That [the rejection] affected us in the way that some – a lot – of the assistants took it personally, as a shortcoming in their professional competence. And it isn’t – but it was [perceived that way] regardless of how you communicated it. So they took it badly. Even if you tried to explain ‘this is not a bad thing, it is just about developing you and your skills and knowledge in terms of how you should do things, so that you can do it.’. But there just wasn’t anyone who heard that.”* (N2)

In that way, the collaboration with the designated GP influenced the interprofessional relations within the care home. Coherently, a nurse reflected that collaboration could depend on the expertise of the social and health care assistant. In the care home where a nurse, a social and health care assistant, and a GP collaborated, the designated GP underscored the importance of knowing the social and health care assistants’ individual competencies and experiences, not just assessing their education alone:

*“Well, we encourage them to go mainly through their nurses if there is something. But it is not always that it … well, you could say, that it has not always proven successful. ... Because there may be a carer or an assistant, who is really accomplished, and who manages the residents well, and all that. And then it will be perfectly fine that she does the writing. So, therefore, it can be difficult to, like say – well, all that matters is education”* (GP3)

Thus, the introduction of a new collaborator (the designated GP) shed light or perhaps intensified existing challenges in the internal and interprofessional collaboration within the care home.

### Interprofessional communication

#### Appropriate and ongoing interprofessional communication

In some cases, the designated GP model was perceived to contribute to a more timely treatment due to more frequent GP visits at the care home and the fact that the care home collaborated with fewer GPs. A nurse elaborated that if she deemed it necessary, it was possible to contact the designated GP on the telephone to for example quickly discuss the patient’s treatment. Before the initiation of the designated GP model, the care home staff often communicated with the GP via a written correspondence message, which could last several days to be answered. A designated GP had a similar experience:

*“So, you may say that [before introducing the designated GP model] I used to have a lot of residents at different care homes, but I did not see them very often because it was a difficult process to go and see them – so then you do really a lot through correspondence and written communication. And we don’t do that quite as much now.”* (GP2)

Thus, the increased frequency of GP visits was experienced to foster a stronger interprofessional relationship which induced emerging issues to be resolved more quickly.

Further, more frequent and in-person communication was experienced to improve interprofessional communication. In the designated GP model, the care home staff and the designated GP explained that they more often exchange and discuss professional experiences related to the treatment of the residents. The care home staff felt that this collaboration improved their professional competencies and knowledge about medical treatment and disease management as a result of an ongoing dialogue. A care home staff member elaborated:

*“And we get more knowledge because we [the care home staff and the designated GPs] have this distinct contact, which allows us to get more knowledge about the residents … ‘why, now I see the same thing as what happened the last time I had a resident presenting like this, and it was that and that and that.”* (N1)

Thus, the dialogue between the care home staff and the designated GPs was an investment that resulted in improved collaboration and thereby improved patient care.

#### Compromises in professional competencies

Although the interprofessional communication was perceived to have improved, it did not always imply that the designated GP and the care home staff agreed on medical decisions. A nurse articulated an acceptance of this:

*“If they [the designated GPs] make an order for a resuscitation, well, then they will get it. I may disagree in it. But that’s how it is – you don’t have to agree on everything.”* (N3)

Improved communication did not necessarily prevent different perceptions of quality of care, which could also lead to professional dissatisfaction. A designated GP explained:

*“Well, the last [patient] I saw, she gets both a morphine patch and Lyrica for pain in her bones – that is, vertebral collapse in her back. And I also tell her that she might easily do without it because it was a long time ago that it happened, the collapse. But she doesn’t want out of it, and she is a little confused. And the staff says, ‘we don’t think so’, and then we don’t go anywhere with it.”* (GP4)

Thus, the increased frequency of GP visits induced by the designated GP model contributed to more appropriate and ongoing communication between the designated GP and the care home staff. However, this communication was not always without disagreement. Both the care home staff and the designated GPs experienced that they sometimes had to compromise their professional competencies in the interprofessional collaboration.

## Discussion

### Principal findings

From the perspectives of care home staff and designated GPs, the designated GP model contributes to more clear, precise, and timely communication between care homes and the GP. In this study, we found that; mutual acknowledgement of each other’s roles and competencies have improved between designated GPs and care home nurses. Sometimes, but not always, this also applied to the relationship between designated GPs and social and health care assistants. Furthermore, we also found that the introduction of a new collaborator, such as the designated GP, sometimes shed light on or perhaps intensified existing challenges in the internal and interprofessional collaboration between nurses and social and health care assistants within the care homes. Professional differences in the interpretation of the patient’s needs were still present, which at times caused a frustrating compromise of own professional competencies. An important reason for the overall perception of an improved collaboration was attributed to the more frequent dialogue (more frequent GP visits at the care homes) in which the care homes staff and the designated GPs exchange knowledge which again could be applied in future patient encounters.

### Comparison with existing literature

In line with our findings, previous research found that a successful collaboration between care home staff and GPs is positively related to quality of care. Studies find that a successful collaboration may positively affect health outcomes and patient safety [25, 26] and that nurses consider interprofessional collaboration valuable when they perceive that their interactions with the GP are beneficial for the residents [27]. Further, Jacobs [28] also found that if only one GP was affiliated with a care home, the interactions could be more beneficial since the GP could build interprofessional relationships with the care home staff, which could lead to improvements in resident care. In addition, a fruitful interprofessional collaboration can result in positive work engagement and job satisfaction [29].

In our study, we found that successful collaboration can be associated with interprofessional understanding of each other’s roles, mutual respect, and knowledge of each other’s professional and personal abilities. Other studies underpin our findings. An understanding of different roles and respect for other professionals is found to be a prerequisite for successful interprofessional collaboration between designated GPs and care home staff [25, 26]. Further, we found that a clarification of the internal collaboration at the care home as well as between the designated GP and care home staff about communication pathways and roles of the GPs and care home staff seems to be important to avoid conflicts and misunderstandings. Common goals for the residents between nurses and GPs proved important to improve communication and collaboration in care homes, including implementing a meeting to establish the common goals and improving accessibility to the GP and care home staff [27]. In a review, Chadborn et al. [30] stated that the arrangement of the relationships between GPs and care home staff was important for the improvement of in prescribing and end-of-life care for residents. Also, the review found that this negotiation about the arrangement was built up over time. Further, a study found that nurses saw the absence of respect from the GPs as failed collaboration but that more mutual respect and knowledge of their profession from the GPs have the ability to improve their relationship with the GPs [27], underscoring the importance of mutual respect and knowledge.

Our results showed that the designated GP model entailed more frequent GP visits at the care homes and that this increased time together seems to play an important role in the improved interprofessional understanding, knowledge and respect, and, consequently, collaboration. Other studies have investigated the importance of regular visits [9, 27, 31]. Regular visits, with subsequent frequent shared informal communication, seem to be associated with improved collaboration between GPs and care home staff members in care homes [27]. Regular communication could include communication about care planning, which may facilitate improvements in access to second opinions and advice, as well as adopted agreements for medication regimes, pressure ulcer treatments etc. [9, 31]. Regular communication in terms of standardized scheduling of GP visits at the care home and improved availability of nurses and GPs has been found to lead to improved collaboration. Furthermore, nurses experienced that their competencies were more valued as the communication improved [27]. Further, a continuous presence of the GP may facilitate a better quality of patient-doctor relations, which will grow over time and result in better knowledge of the patient’s situation and preferences [9].

Thus, in line with other research findings, we have identified a complex interplay between quality of care, interprofessional collaboration, communication, understanding of each other’s roles, mutual respect, and knowledge of each other’s professional and personal abilities. The importance of frequency of GP visits identified in others as well as our study underlines that a major reason for the improved collaboration in the Danish designated GP model is related to gathering GPs at specific care homes, which results in more frequent visits, in order for an improved interprofessional understanding, respect, and knowledge to take place.

### Implications

In view of the ageing population, a useful designated GP model will continue to be needed. Our results illustrate that the designated GP model holds the potential to facilitate improved communication and, in that way, improved quality of care. Moreover, our results could also serve as a knowledge base to generate a deeper understanding of the care home staff and designated GPs’ perspectives, roles, and knowledge, for further development of the model. The designated GP model can support closer collaboration and constructive discussions between care home staff and GPs, which could lead to better knowledge and a greater understanding of each other. An examination of the views and wishes of other stakeholders involved in the designated GP model, namely residents and relatives, could illuminate the model further. These will complete the detailed depth view of the model and ultimately facilitate better collaboration in care homes and improve the residents’ care.

#### Strengths and limitations

To our knowledge, this is the first study to qualitatively explore designated GPs and care home staff’s experiences of their team-based collaboration. The principal strength of this study was the inclusion of designated GPs and care home staff (nurses and a social and health care assistant), who actively work together in a team, and who are relevant stakeholders in a care home setting representing different perceptions of quality [32]. Another strength included the sampling strategy to obtain a maximum variation of care homes representing different ways of organising the designated GP model. By interviewing both GPs and staff, the interview topics could be explored from different perspectives, thereby increasing the credibility of the study [22]. Moreover, additional background information about the participants could have been preferable, since age and years of work experience could potentially influence the perspectives on interprofessional collaboration. Further, using the theory of relational coordination [23] together with our reflections and discussions during the analysis raised awareness of possible biases and preconceived assumptions about what could be found in the study, which strengthens the trustworthiness. Also, the researchers had different educational backgrounds and research experiences which further strengthened the reliability.

This study has several limitations. First, an acknowledged limitation was that the study only contained data from interviews, and thus the actual collaborative practice was not observed. Other collaborative practices, e.g. tacit, implicit behaviours may exist in the collaborative practices, which an observational study may have discovered. Further, the study was performed in a Danish care home setting and the organization of the Danish health care system may vary from other health care systems. Second, the data focused on perspectives from a specific urban setting. For example, GPs and care home staff in rural settings with a lower number of GPs in the same area might experience a smaller difference after the introduction of the designated GP model, compared to an urban setting. Also, as small differences in the organization of the designated GP model exist across municipalities, the inclusion of informants from other municipalities may have illuminated other perspectives on the collaboration within the designated GP model, as would have the inclusion of a higher number of informants.

## Conclusion

Designated GPs and care home staff experienced that their interprofessional team-based collaboration had improved as a result of the designated GP model. The improved collaboration was attributed to the more frequent dialogue with exchanges of patient-related knowledge and mutual acknowledgement of each other’s roles and competencies. Even though the current designated GP model is a good starting point, the results call for a more clear and more straightforward way to communicate between GPs and care home staff about patient-related issues.

## Supplementary Information

**Additional file 1.** Consolidated criteria for reporting qualitative studies (COREQ): a 32-item checklist.

**Additional file 2.** Interview guides for semi-structured interviews with GPs and care home staff.

## Data Availability

Anonymised interview transcripts can be made available from the corresponding author upon reasonable request.

## Abbreviation

COREQ: Consolidated criteria for reporting qualitative research; GP: General practitioner
