# Supplementary material for: Interprofessional team-based collaboration between designated GPs and care home staff: a qualitative study in an urban Danish setting
Source: BMC Prim Care. 2023 Jan 4;24:3. doi: 10.1186/s12875-023-01966-1 (PMC9811752; doi:10.1186/s12875-023-01966-1)
Supplement: Supplementary file 1 — Additional file 1. Consolidated criteria for reporting qualitative studies (COREQ): a 32-item checklist. [file 12875_2023_1966_MOESM1_ESM.docx]

Additional file 1

**Consolidated criteria for reporting qualitative studies (COREQ): a 32-item checklist**

| **Domain 1: Research team and reﬂexivity** | | |
| --- | --- | --- |
| *Personal characteristics* | | |
| Interviewer/facilitator | 1 | Line Due Christensen (see page 8) |
| Credentials | 2 | PhD |
| Occupation | 3 | Pharmacist, postdoc |
| Gender | 4 | Female (see title page) |
| Experience and training | 5 | Previously carried out qualitative research, including semi-structured interviews and focus group interviews in both hospital and community settings. Feedback on the interviews by AM, who is an experienced qualitative researcher. |
| *Relationship with participants* | | |
| Relationship established | 6 | Initially, LDC contacted the care homes and the GPs by telephone or email to explain about the study. No relationship with the participants existed before the interviews. All participants were informed by LDC that participation in the study was voluntary. All informants gave written informed consent. GPs were remunerated for their participation. No payment was given to the care home staff for their participation (see page 6 + 19). |
| Participant knowledge of the interviewer | 7 | The interviewer was presented as a researcher interested in the collaboration between general practices and care homes. |
| Interviewer characteristics | 8 | No characteristics were reported. |
| **Domain 2: Study design** | | |
| *Theoretical framework* | | |
| Methodological orientation and Theory | 9 | We conducted a descriptive qualitative study, using semi-structured interviews to gain insights into designated GPs’ and care home staffs’ perspectives of their mutual interprofessional collaboration. We used content analysis with inspiration from the theory of relational coordination [14]. To secure the quality and trustworthiness of the study, we followed the main domains and each sub-item presented in the checklist: The Consolidated criteria for reporting qualitative research (COREQ) 32-item checklist (Additional file 1) [15] (see page 5). |
| *Participant selection* | | |
| Sampling | 10 | The care homes were purposefully selected in order to include care homes with a maximum variation regarding the organisation of the collaboration between care homes and the designated GP (see page 6) |
| Method of approach | 11 | Participants were asked for participation by LDC either via telephone or email (see page 6). |
| Sample size | 12 | 11 participants (see page 7) |
| Non-participation | 13 | None refused to participate (see page 7) |
| *Setting* | | |
| Setting of data collection | 14 | Care homes and general practice (see page 8) |
| Presence of non-participants | 15 | None |
| Description of sample | 16 | The care home staff were from four different care homes, and the GPs were affiliated with each of the care homes. Ten participants were female and one participant was male (see page 7+8). |
| *Data collection* | | |
| Interview guide | 17 | The interview guide was semi-structured and had open-ended questions, based on the theory of relational coordination (see page 8). The interview guide was not piloted, but was revised continuously throughout the process of data collection. |
| Repeat interviews | 18 | No interviews were repeated. |
| Audio/visual recording | 19 | Interviews were audio-recorded. The transcription was carried out by a research assistant, and the transcripts were checked for fidelity according to the audio records by LDC (see page 8). |
| Field notes | 20 | LDC wrote field notes immediately after each interview (see page 8). |
| Duration | 21 | The interviews had a duration of 24-55 minutes (see page 8) |
| Data saturation | 22 | In order to iteratively guide an adequate sample size, we used the concept of information power suggesting that the more information the sample holds, the lower number of participants is required. After interviews with care home staff and GPs from four different care homes, we made an iterative interpretative judgement that there was sufficient information power to address study aims. In this process, we considered the study aim, sample specificity, use of established theory, analysis strategy, and quality of dialogue. Our study aim was narrow due to a research question which was highly focused on the experience of the interprofessional team-based collaboration between the designated GPs and care home staff in Danish care homes. The participants were selected using purposive sampling for characteristics highly specific to the study aim. The theory of relational coordination informed both the interview guide and the analysis. As the interviewer was a researcher with previous qualitative experiences and the interview guide was developed by multiple rounds of discussions in the research group, we estimated the quality of dialogue to be sufficient (see page 6+7). |
| Transcripts returned | 23 | No transcripts were returned to participants for comments. |
| **Domain 3: analysis and ﬁndings** | | |
| *Data analysis* | | |
| Number of data coders | 24 | One (LDC). All authors engaged in ongoing discussions about and agreed upon the emergent themes (see page 8). |
| Description of the coding tree | 25 | Once the coding process was complete, codes with mutual characteristics were grouped into emergent themes that were finally assembled into the overall themes of the theory of relational coordination and the overall aim of the study (see page 8+9). |
| Derivation of themes | 26 | Data were analysed both inductively and deductively in the process (see page 8+9). |
| Software | 27 | No software was used to manage the data (see page 9). |
| Participant checking | 28 | Participants did not provide feedback on the findings. |
| *Reporting* | | |
| Quotations presented | 29 | In order to illustrate the findings, quotations are presented in the paper together with the identification of the participant (see page 9-14). |
| Data and ﬁndings consistent | 30 | There is consistency between the data presented in the paper and the findings (see page 9-14). |
| Clarity of major themes | 31 | Major themes (main themes) are clearly presented in the paper. (see page 9-14). |
| Clarity of minor themes | 32 | Minor themes (subthemes) are clearly presented in the paper (see page 9-14). |

Reference: Tong A, Sainsbury P, Craig J. Consolidated criteria for reporting qualitative research (COREQ): a 32-item checklist for interviews and focus groups. Int J Qual Health Care. 2007, 19(6):349-357.
