# Supplementary material for: Interprofessional team-based collaboration between designated GPs and care home staff: a qualitative study in an urban Danish setting
Source: BMC Prim Care. 2023 Jan 4;24:3. doi: 10.1186/s12875-023-01966-1 (PMC9811752; doi:10.1186/s12875-023-01966-1)
Supplement: Supplementary file 2 — Additional file 2. Interview guides for semi-structured interviews with GPs and care home staff. [file 12875_2023_1966_MOESM2_ESM.docx]

Additional file 2

### Interview guide for semi-structured interviews with GPs

| **Themes** | **Questions*** |
| --- | --- |
| **1. Background** **information** | |
|  | - 1. Can you briefly describe your workplace (type of general practice etc.)?   2. What were your considerations/motivations to become a designated GP?   3. Can you describe how many and what type of residents you have in the care home? |
| **2. Tasks** | |
|  | - 1. Could you describe what you do as a designated GP?   2. Do you feel that you have enough time for your job as a designated GP?   3. Can you describe when your tasks at the care home go well / less well? |
| **3. Significance of the designated GP model on medication** | |
|  | - 1. Do you experience any changes in the use of medication among the residents after you became a designated GP at the care home? If yes, please elaborate. |
| **4. Teaching** | |
|  | - 1. Do you teach the care home staff? Can you elaborate on that? |
| **5. Collaboration** | |
| Based on Judy Gittell [23] | - 1. How would you describe the collaboration between you and the care home?   2. How do you share knowledge (between physicians/care home staff and designated GP)?   3. Do you and the care home staff have a clear division of tasks? Can you elaborate on that?   4. Have you and the care home staff evaluated the collaboration? Can you elaborate on that?   5. Do you feel that you see the same goal for the residents? Can you elaborate on that?   6. Do you think that the care home staff lacks knowledge about medicine/treatment of the elderly / understanding of illness? Can you elaborate on that?   7. Do you feel that the care home staff has selected the residents who most need your help at your regular visits? Why / why not   8. Do the care home staff contact you with relevant resident requests? In time? Can you elaborate on that?   9. How have you experienced the quality of the requests?   10. How have you experienced resident-related issues being resolved at the care home? |
| **6. Recommendations** **to improve the collaboration** | |
|  | - 1. What would you recommend to other GPs who are going to begin as designated GP at a care home? Why / why not |

### Interview guide for semi-structured interviews with care home staff

| **Themes** | **Questions*** |
| --- | --- |
| **1. Background** **information** | |
|  | - 1. Can you briefly introduce yourself by name and your educational background?   2. For how long have you the designated GP been affiliated with the care home?   3. How often does the designated GP visit the care home?   4. For how long have you worked together with the designated GP? |
| **2. Tasks** | |
|  | - 1. Can you describe your tasks in connection with the designated GP?   2. Do you have any thoughts on why you have these tasks? Have the tasks changed since you began collaborating with the designated GP?   3. Do you feel that you have enough time for the tacks? Can you elaborate on that?   4. Does the designated GP have enough time for the residents? Why/why not?   5. Can you describe when your tasks at the care home go well / less well?   6. Can you tell me about the correspondence messages between the care home and the designated GP? Too many?   7. If you could choose, do you want other tasks? Why? |
| **3. Teaching** | |
|  | - 1. Have you experienced teaching from the designated GP? If yes, what are your experiences with that?   2. Do you need more teaching? |
| **4. Significance of the designated GP model on medication** | |
|  | - 1. Have you experienced the use of medication among the residents changed since the designated GP model was introduced? and how? |
| **5. Collaboration** | |
| Based on Judy Gittell [23] | - 1. How would you describe the collaboration between you and the designated GP? Is collaboration with the designated GP easier than with other GPs?   2. Can you elaborate on the communication between you and the designated GP?   3. How do you share knowledge (between care home staff/care home staff and designated GP)?   4. Do you and the designated GP have a clear division of tasks? ? Can you elaborate on that?   5. Have you evaluated the collaboration? Can you elaborate on that?   6. Do you feel that you (the designated GP and care home staff) see the same goal for the residents? Can you elaborate on that?   7. Do you feel that the designated GP is responsive to the resident-related issues you experience? Can you elaborate on that?   8. How have you experienced resident-related issues being resolved? |
| **6. Recommendations** **to improve the collaboration** | |
|  | - 1. What recommendations do you have for other care homes that are going to work together with a new designated GP (or where the collaboration does not work)? |

*Follow-up questions:

1. What does it mean when you say…?
2. Can you explain/tell me more? What happened? What did you do?
3. Can you provide a specific example?

[23] Gittell JH, Godfrey M, Thistlethwaite J. Interprofessional collaborative practice and relational coordination: improving health care through relationships. *J Interprof Care*. 2013 May; 27: 210-3.
